# Depth Imaging-Based Framework for Efficient Phenotypic Recognition in Tomato Fruit

**DOI:** 10.3390/plants14223434

**Published:** 2025-11-10

**Authors:** Junqing Li, Guoao Dong, Yuhang Liu, Hua Yuan, Zheng Xu, Wenfeng Nie, Yan Zhang, Qinghua Shi

**Affiliations:** 1 College of Information Science and Engineering, Shandong Agricultural University, Tai’an 271018, China; junqing.li@sdau.edu.cn (J.L.); d_guoao@163.com (G.D.); 17661290553@163.com (H.Y.); xbzz101800@163.com (Z.X.); 2 College of Horticulture Science and Engineering, Shandong Agricultural University, Tai’an 271018, China; 13210693446@163.com (Y.L.); nwf2024@sdau.edu.cn (W.N.); zhangyan2022@sdau.edu.cn (Y.Z.)

**Keywords:** tomato fruit phenotyping, phenotypic recognition, deep learning, depth imaging, phenotypic analysis

## Abstract

Tomato is a globally significant horticultural crop with substantial economic and nutritional value. High-precision phenotypic analysis of tomato fruit characteristics, enabled by computer vision and image-based phenotyping technologies, is essential for varietal selection and automated quality evaluation. An intelligent detection framework for phenomics analysis of tomato fruits was developed in this study, which combines image processing techniques with deep learning algorithms to automate the extraction and quantitative analysis of 12 phenotypic traits, including fruit morphology, structure, color and so on. First, a dataset of tomato fruit section images was developed using a depth camera. Second, the SegFormer model was improved by incorporating the MLLA linear attention mechanism, and a lightweight SegFormer-MLLA model for tomato fruit phenotype segmentation was proposed. Accurate segmentation of tomato fruit stem scars and locular structures was achieved, with significantly reduced computational cost by the proposed model. Finally, a Hybrid Depth Regression Model was designed to optimize the estimation of optimal depth. By fusing RGB and depth information, the framework enabled efficient detection of key phenotypic traits, including fruit longitudinal diameter, transverse diameter, mesocarp thickness, and depth and width of stem scar. Experimental results demonstrated a high correlation between the phenotypic parameters detected by the proposed model and the manually measured values, effectively validating the accuracy and feasibility of the model. Hence, we developed an equipment automatically phenotyping tomato fruits and the corresponding software system, providing reliable data support for precision tomato breeding and intelligent cultivation, as well as a reference methodology for phenotyping other fruit crops.

## 1. Introduction

Tomato (*Solanum lycopersicum* L.) is a globally significant horticultural crop with high economic and nutritional value, containing functional substances, such as vitamin C and lycopene [1,2]. Modern cultivated tomatoes originated from the red-fruited wild species (*Solanum pimpinellifolium*) through domestication and selection for traits such as fruit size and sugar accumulation [3]. In recent years, advances in breeding technologies have resulted in a diversification of tomato varieties, significantly enriching the available germplasm resources [4,5]. Phenotypic characteristics of tomato fruits, including shape, color and texture, directly influence their commercial grading, mechanized harvesting, and post-harvest quality [6]. For instance, traditional soft-textured tomato varieties are unsuitable for mechanical harvesting, prompting breeding efforts toward firmer textures [7]. The locular gel (a gelatinous tissue within fruit locules) in tomato fruits constitutes the second most abundant tissue after the pericarp, accounting for approximately 23% of the fruit’s fresh weight [8]. Research has demonstrated that the size and number of locules affect fruit weight, shape, and texture, and to some extent influence flavor and mouthfeel [9,10]. In tomato breeding, traits such as locule number, mesocarp thickness, transverse and longitudinal diameters, fruit shape index, and color are emphasized by breeders. Additionally, the depth and width of the stem scar in the fruit are identified as important factors affecting the appearance and commercial value of tomatoes.

Underlying genetic regulatory mechanisms of tomato fruit traits, including size, shape, color, taste and nutritional content, have emerged as key research foci in plant science [11,12,13,14]. Modern tomato breeding strategies have transitioned from phenotypic selection to integrated genotype-phenotype prediction [15]. This shift has made the acquisition of high-throughput phenomics data a critical step in tomato breeding. Current phenotyping relies heavily on manual measurements, which are subjective, inefficient, and unscalable for high-throughput breeding. Through in-depth studies, researchers have achieved significant breakthroughs in understanding the genetic basis and regulatory mechanisms of tomato development [16,17,18]. However, the morphological diversity of tomato fruits, such as locule number ranging from 3 to 10, and their developmental dynamics pose substantial challenges. In this context, the development of high-precision and rapid techniques for identifying and measuring tomato fruit phenotypes is essential for the quantitative analysis of fruit traits. Such techniques also provide high-frequency dynamic phenomics inputs for genomic selection models, which are significant for enhancing the efficiency of tomato fruit improvement and establishing robust fruit evaluation models.

In recent years, innovations in artificial intelligence technology have injected strong momentum into crop phenomics. Notably, the integration of computer vision and deep learning has significantly enhanced the automated parsing capabilities of phenotypic parameters [19,20,21], providing technical support for efficient breeding decisions. In the field of fruit detection, Zhang et al. [22] proposed a lightweight fruit-detection algorithm using Light-CSPNet as the backbone network. By incorporating improved feature extraction modules, downsampling methods, and feature fusion modules, they achieved real-time detection of tomato fruits on edge devices while maintaining detection accuracy. For the task of winter jujube detection, Yu et al. [23] introduced an MLG-YOLO model based on YOLOv8n, which achieves three-dimensional localization of winter jujubes by integrating an RGB-D camera. In the field of maturity assessment, Wan et al. [24] combined characteristic color values with backpropagation Neural Network (BPNN) technology to detect the maturity of commercially available tomatoes. They constructed a quantitative model for tomato fruit maturity based on threshold segmentation and concentric sub-region division strategies. In the field of commercial grading, Ireri et al. [25] proposed a machine vision system for tomato grading based on RGB images, which accurately identified abnormalities in calyx morphology and fruit scars. In the field of crop disease diagnosis, Deng et al. [26] proposed an image-based method for segmenting tomato leaf diseases, MC-UNet, which employs a multi-scale convolution module to capture multi-scale information of tomato diseases. By utilizing a cross-layer attention fusion mechanism with gating structures and fusion operations, they effectively improved the boundary consistency of leaf spot segmentation. Kang et al. [27] improved the deep learning network YOLO-TGI by integrating Ghost and CBAM modules to assess the health status of tomato leaves and simultaneously count tomatoes in video streams. These technologies collectively demonstrate the technical feasibility of automated extraction of fruit phenotypes.

The key to accurate extraction of fruit phenotypic characteristics is the precise assessment and quantification of multidimensional data such as the transverse diameter, longitudinal diameter, and color of the fruit [28]. Zhu et al. [29] proposed an automatic detection method for tomato fruit phenotypes based on image recognition, using the Mask R-CNN model to train and test the structural indicators of tomato fruit locules. They extracted the color, transverse and longitudinal diameters, top and navel angles, locule number, and pericarp thickness of tomato fruits, achieving automated measurement of multiple phenotypic parameters. Xu et al. [30] successfully extracted 11 phenotypic traits, such as melon size, pedicel, and color, by constructing a deep learning algorithm framework, and R^2^ values were above 0.94 for fruit transverse diameter and 0.698 for fruit longitudinal diameter. Xue et al. [31] proposed a cucumber fruit morphological trait recognition framework and software called CucumberAI, which integrates deep learning algorithms and can effectively identify 51 cucumber features, and achieved R^2^ values greater than 0.9 for fruit diameter, neck length, and fruit length. These phenotype extraction methods, based on image recognition and deep learning, provide new technical approaches for the precise analysis of fruit phenotypic traits.

When obtaining the size of tomato fruits, traditional methods often rely on introducing a reference object in the image and inferring the actual size of the fruit by comparing the size ratio of the fruit to the reference object in the image. However, this method has significant limitations: to ensure measurement accuracy, the reference object and the fruit should ideally be on the same plane. Considering that tomato fruits vary in size and grow in different positions, it is difficult to ensure that all fruits and the reference object are on the same plane, thus easily introducing measurement errors. With the rapid advancement of perceptual imaging technology and the widespread adoption of consumer-grade RGB-D cameras, real-time depth estimation combined with RGB imaging can be achieved at low cost. This significantly enhances remote non-contact sensing capabilities, continuously improves measurement accuracy, and greatly boosts environmental adaptability. These devices mostly use the time-of-flight (TOF) principle for ranging, which not only provides 2D information of the target object but also the depth value of each pixel in the image, providing rich data information for the study of crop morphological characteristics [32,33,34]. Due to their convenience and practicality, RGB-D cameras are increasingly recognized by researchers and agricultural practitioners, and are gradually becoming important tools for detecting and extracting crop morphological characteristics.

For high-throughput automatic phenotypic analysis of tomato fruit traits, in this study, a more comprehensive and efficient intelligent phenotyping framework was developed. The contributions are summarized as follows:

1. The framework analyzes 12 phenotypic traits, including fruit transverse and longitudinal diameters, shape index, stem scar structure in the fruit, as well as stem scar depth and width, locule structure, locule number, locule area, mesocarp thickness, mesocarp color, and locule color.
2. Based on the SegFormer architecture [35], the MLLA linear attention mechanism was introduced to develop a SegFormer-MLLA model for tomato fruit phenotypic traits segmentation [36]. This model enhances computational efficiency while maintaining high segmentation accuracy, enabling precise segmentation of the locule and stem scar structures in tomato fruits.
3. By integrating depth information, the dimensions of tomato fruit traits were measured. To address depth information errors caused by optical interference, such as specular reflections, a Hybrid Depth Regression Model (HDRM) was designed. This model captures the optimal depth distance of the tomato fruit images through modeling parameter errors, calculating residuals, and applying random forest-based residual correction.
4. We designed an intelligent detection system for tomato fruit phenomics analysis, which integrates both software and hardware components. During the detection process, each sample was assigned a corresponding label to establish a mapping with its phenotypic data, enabling efficient and accurate detection and data storage of tomato fruit phenotypic traits.

## 2. Materials and Methods

### 2.1. Image Acquisition

**Figure 1:**
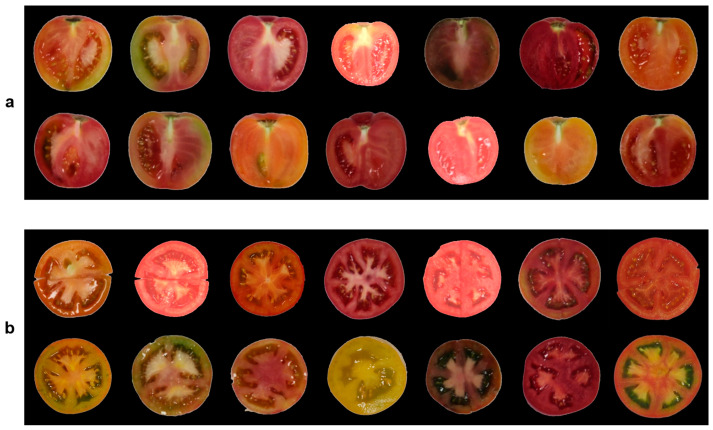
Examples of tomato fruit section images. (**a**) Tomato fruit longitudinal section images; (**b**) Tomato fruit transverse section images.

Tomato fruit samples used in this study were collected from Shandong Agricultural University Science and Technology Innovation Park (36.163° N, 117.165° E) and Taian Hengchang Ecological Agriculture Company (36.221° N, 116.864° E). The tomato varieties used in this study were previously collected materials in our laboratory. A total of 322 mature tomato fruits, representing multiple varieties and colors, were randomly selected. For longitudinal sections, the fruits were cut along the central axis passing through the stem scar. For transverse sections, the fruits were cut through the fruit center. The resulting sections were placed on a uniform white background, and high-resolution images of both longitudinal and transverse sections were captured using an Azure Kinect 3.0 depth camera under consistent illumination conditions. The depth camera was fixed using a top-view bracket, and images were captured at a resolution of 3840 × 2160 pixels. Some examples of the tomato fruit images are shown in Figure 1, illustrating detailed features of the stem scar and locules. Specifically, considerable variation was observed in the tomato for the number of locules (2–10 per fruit), along with the stem scar, and the shape and size of the locules.

### 2.2. Image Preprocessing

To eliminate background noise interference in the tomato section images and unify the data scale, an image preprocessing workflow was designed, which consists of four main modules: binarization, image cropping, data annotation, and data partitioning.

1. Binarization module

**Figure 2:**
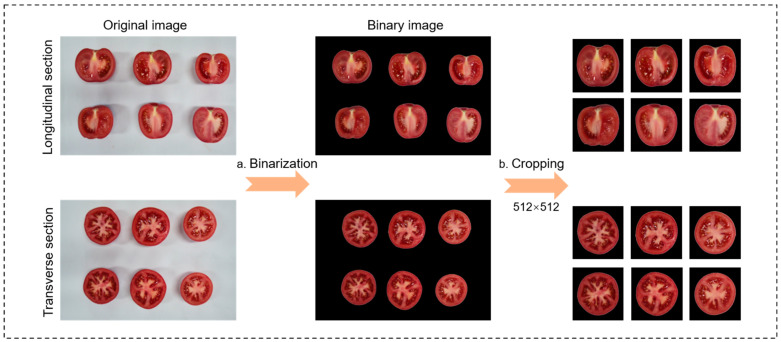
Preprocessing workflow for tomato fruit section images. (**a**) Binary thresholding of tomato fruit images to remove background interference; (**b**) Cropping of tomato fruit images to 512 × 512 pixels.

The binarization process is shown in Figure 2a. Tomato fruits were extracted based on the HSV color space, and noise interference was reduced using closing and opening operations to enhance image quality. Due to color differences in tomatoes and interference from the stem scar color domain, the extracted tomatoes exhibited some pixel loss. To address this issue, Canny edge detection [37] combined with contour extraction was employed to identify the edges of the tomato fruits. After removing noise through opening operations and multiple rounds of erosion, the image binarization process was completed.

2. Image cropping module

The image cropping process is illustrated in Figure 2b. To ensure consistency in the size of tomato section images and to meet the requirements for model training, all image samples were cropped and resized to 512 × 512 pixels, resulting in individual tomato fruit section images.

3. Data annotation module

The LabelMe tool was employed to annotate longitudinal and transverse sections of tomato fruits with distinct strategies: longitudinal sections were annotated along the stem scar contour, while transverse sections were marked along locule contours, including irregular locule structures. Annotated data were then converted into JSON files and formatted for model training.

4. Data partitioning module

For the purpose of model training and validation, the dataset was randomly split into training, validation, and test subsets in a 7:2:1 ratio. To prevent potential data leakage, the training, validation, and test datasets were stored in separate directories. For data augmentation, the training set was subjected to a combination of cropping, scaling, rotation, and brightness adjustment to enhance sample diversity, while the validation and test sets retained only cropping, scaling, and rotation to preserve the original data characteristics. After data augmentation, a total of 8392 tomato section images were obtained.

### 2.3. SegFormer-MLLA Model

SegFormer [35] is a semantic segmentation model based on Transformer [38], consisting of an encoder and a decoder. The encoder is constructed with four layers of transformer blocks, each stage employing downsampling rates of 4, 8, 16, and 32, respectively, to progressively extract multi-scale features. Each transformer block includes an efficient self-attention module, a Mix-FFN (mixed feed-forward network) module, and an overlapped patch merging module to efficiently capture both global and local features. The four feature maps of different resolutions from the encoder layers are fused in the decoder after passing through an MLP layer, and then the prediction mask is obtained after another MLP. Unlike traditional segmentation models with complex decoder architectures, SegFormer utilizes a lightweight all-MLP decoder, effectively reducing computational overhead and simplifying parameter tuning.

The standard self-attention mechanism based on the Transformer architecture has a quadratic computational complexity of O(*N*^2^), which poses bottlenecks in terms of computational resources and memory usage. SegFormer employs a hierarchically structured Transformer encoder architecture combined with efficient sequence reduction techniques, lowering the time complexity from O(*N*^2^) to O(*N*^2^/*R*). Nevertheless, as its core is still based on an inherently quadratic-complexity attention mechanism, the overall optimization is limited. Early linear attention mechanisms replaced the nonlinear Softmax function with linear normalization, reducing the computational complexity to O(*N*). However, due to limitations in feature representation capability, their actual performance still lags behind traditional attention mechanisms. Recently, the Mamba architecture based on state space models (SSMs) has achieved significant development in the field of image segmentation [39,40,41], as it maintains linear computational complexity while establishing long-range dependencies. MLLA [36] combines the selective mechanism of Mamba with the advantages of linear attention, achieving parallel processing efficiency for attention and adaptive feature selection capability of SSM, thereby enabling efficient image processing with O(*N*) complexity. MLLA Attention employs RoPE positional encoding [42] instead of a forget gate, providing necessary positional information while maintaining parallel computation and fast inference speed, thereby overcoming the limitations of recursive computation. By combining LePE positional encoding [43] with RoPE positional encoding, it can flexibly handle features at different scales.

**Figure 3:**
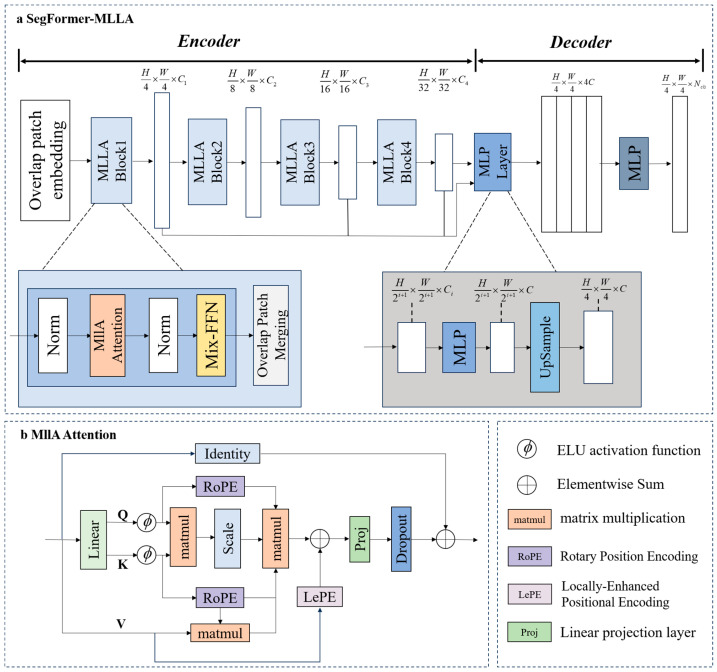
Schematic diagram of the SegFormer-MLLA model structure. (**a**) Overall architecture of SegFormer-MLLA, incorporating MLLA blocks to replace transformer blocks for improved feature extraction efficiency; (**b**) MLLA attention module, designed to capture both global and local features, enhancing the precision of tomato phenotypic trait analysis.

To achieve efficient and precise segmentation of tomato fruit stem scar and locule structures, we proposed the SegFormer-MLLA model for tomato fruit phenotyping, which optimizes the encoder layer based on SegFormer and introduces the MLLA linear attention mechanism. The overall architecture of SegFormer-MLLA is shown in Figure 3a. To enhance the generalization ability of MLLA, a linear projection layer (*Proj*) is used to map features to a unified embedding dimension, thereby improving feature representation, and dropout is combined for regularization to reduce the risk of overfitting.

The MLLA Attention module, which is the key feature extraction component of the model, has an overall architecture as shown in Figure 3b and aims to capture long-range and local dependencies in images with linear complexity. The projections of the query (*Q*), key (*K*), and value (*V*) vectors are processed through activation functions and then realized via linear transformations. The formula for calculating the attention output of MLLA Attention is given in Equations (1) and (2):

(1) $$Q=\phi (xW_{Q}),\;K=\phi (xW_{K}),\;V=xW_{V}$$

(2) $$y_{i}=\sum_{j=1}^{N}\frac{Q_{i}K_{j}^{⊤}}{\sum_{j=1}^{N}Q_{i}K_{j}^{⊤}}V_{j}=\frac{Q_{i}(\sum_{j=1}^{N}K_{j}^{⊤}V_{j})}{Q_{i}(\sum_{j=1}^{N}K_{j}^{⊤})}$$

where *ϕ* denotes the additional kernel function introduced, *x* denotes the input, and *W_Q_*, *W_K_*, and *W_V_* denote the learnable weight matrices for the *Q*, *K*, and *V* projections, respectively, with each *Q* aggregating information from all *K* and *V*.

(3) $$y_{i}=\frac{Q_{i}S_{i}}{Q_{i}Z_{i}},\;S_{i}=\sum_{j=1}^{i}K_{j}^{⊤}V_{j},\;Z_{i}=\sum_{j=1}^{i}K_{j}^{⊤}$$

This further derives cyclic linear attention, as expressed in Equation (4).

(4) $$S_{i}=S_{i-1}+K_{i}^{⊤}V_{i},\;Z_{i}=Z_{i-1}+K_{i}^{⊤},\;y_{i}=\frac{Q_{i}S_{i}}{Q_{i}Z_{i}}$$

RoPE [42] uses rotational position embeddings to provide global position information for Q and K, allowing the attention mechanism to capture relative positional dependencies. LePE [43] uses depthwise convolution to extract local spatial features for V. This mechanism acts like a forget gate, which enhances the model’s fine-grained spatial perception. MLLA Attention integrates RoPE and LePE to effectively fuse global and local information [40], as described below:

(5) $$RoPE(x_{m},\theta_{i})=x_{m}\cdot (\cos(m\theta_{i})+\sin(m\theta_{i}))$$

(6) $$LePE(x)=x+DWConv(x)W_{L}$$

(7) Attn=(RoPE(Q), RoPE(K), V+LePE(V))

where *m* denotes the dimensionality of the input *x*, *θ_i_* denotes the position-related angle, *W_L_* denotes the learnable weight matrix, and *DWConv* denotes a depthwise convolution with a kernel size of *k*.

### 2.4. Tomato Fruit Phenotypic Size Transformation

Based on the acquired phenotypic parameters of tomato fruit sections, this study utilizes depth information to convert pixel distances into real-world physical distances. Specifically, RGB image pixels were remapped through distortion correction to generate an undistorted image. The depth image records the actual depth value d of each pixel from the camera. By integrating the camera’s intrinsic matrix *K* with the depth value *d*, a mapping relationship from pixel coordinates (*u*, *v*) to three-dimensional physical coordinates (*X*, *Y*, *Z*) was established, as given by Equations (8) and (9):

(8) $$K=\left|\begin{array}{ccc} f_{x} & 0 & c_{x}\\ 0 & f_{y} & c_{y}\\ 0 & 0 & 1 \end{array}\right|$$

(9) $$\left(x,y,z)=(\frac{(u-c_{x})\cdot d}{f_{x}},\frac{(v-c_{y})\cdot d}{f_{y}},d\right)$$

where *K* denotes the intrinsic matrix, *f_x_* and *f_y_* represent the focal length parameters, (*c_x_*, *c_y_*) indicates the principal point coordinates, and *d* is the raw distance value obtained from the depth sensor.

**Figure 4:**
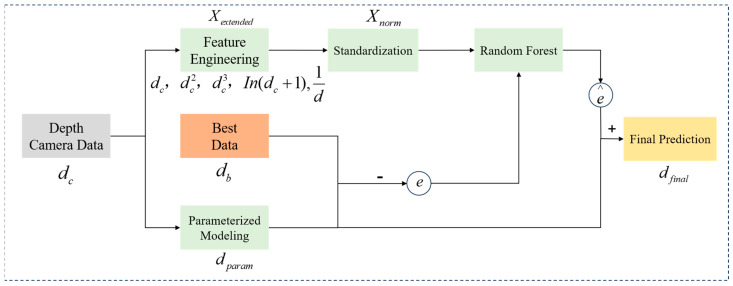
Schematic diagram of the HDRM model structure. The model is trained using captured depth information and optimal depth information to generate final prediction results.

A preliminary analysis of the phenotypic data from tomato fruit sections revealed deviations in both fruit depth values and measured dimensions. The potential causes are hypothesized to include the following: (1) the specular reflection effect, where the smooth surface of the tomato fruit sections causes specular reflection of structured light, disrupting phase computation; (2) the light scattering effect due to tissue moisture, where water-rich tomato tissues and cellular structures induce multiple light scattering, resulting in distortion of light distribution and errors in depth estimation; (3) multipath errors, where positional variations in multiple tomatoes in the image lead to depth estimation errors. To address these issues, a Hybrid Depth Regression Model (HDRM) was developed to fit the optimal depth distance, with its core process depicted in Figure 4.

The key modules illustrated in Figure 4 are designed as follows:

1. Parameterized Modeling for Error Correction. A nonlinear parametric model was established, and the optimal parameter solution was obtained by fitting the objective function using the least squares method. Let *d_c_* denote the depth value measured by the camera, *d_b_* denote the optimal depth value, which is obtained through multiple rounds of tuning and calibration, and *d_param_* denote the initial predicted depth value as given by Equation (10):
  (10) $$d_{param}=(1+\alpha )d_{c}+\beta d_{c}^{2}+\phi +\frac{\gamma }{d_{c}}$$
  where *α*, *β*, *ϕ*, and *γ* are parameters derived from data fitting.
2. Residual Calculation. The residual error *e*, which denotes the deviation predicted by the parametric error model, was calculated for further correction using a random forest regression model, as defined below:
  (11) $$e(d)=d_{b}-d_{param}$$
3. Feature Engineering. To further capture the nonlinear relationships within the residuals, an extended feature set *X_extended_* was constructed, including linear, quadratic, cubic, logarithmic, and reciprocal terms:
  (12) $$X_{extended}=[d_{c},d_{c}^{2},d_{c}^{3},\ln(d_{c}+1),\frac{1}{d}]$$
4. Standardization. To prevent feature scale discrepancies from affecting model training, the feature set was standardized to obtain *X_norm_*, ensuring consistency in the input to the random forest model. Here, *μ* denotes the mean of the feature vector, and *σ* denotes the standard deviation of the feature vector:
  (13) $$X_{norm}=\frac{X_{extended}-\mu }{\sigma }$$
5. Random Forest Regressor for Residual Correction (*RF*). A random forest *RF* model was employed to predict the residual correction value $\overset{\wedge }{e}$. The *RF* model integrates the outputs of multiple decision trees, *T_k_*, and its predicted value is given by:
  (14) $$\overset{\wedge }{e}(d)=RF(X_{norm})$$
6. Final Corrected Model Depth. The final corrected depth is the sum of the parametric model prediction and the random forest residual prediction:
  (15) $$d_{fianl}=d_{param}+\overset{\wedge }{e}(d)$$

### 2.5. Tomato Fruit Phenotype Recognition Process

**Figure 5:**
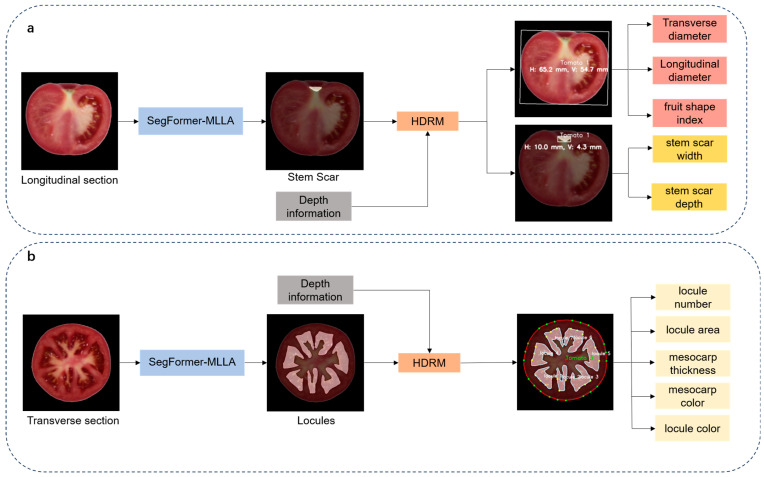
Workflow for detecting phenotypic traits of tomato fruit. (**a**) Phenotypic detection of longitudinal sections; (**b**) Phenotypic detection of transverse sections.

By integrating image processing techniques with deep learning algorithms, this study analyzed tomato fruits from both longitudinal and transverse sectional views and proposed a framework for the automated identification of phenotypic traits, as shown in Figure 5.

The framework enables high-throughput and precise extraction of multi-dimensional phenotypic traits from tomato fruits of various varieties, including transverse and longitudinal diameters, fruit shape index, stem scar structure, stem scar depth and width, locule structure, locule number, locule area, mesocarp thickness, mesocarp color, and locule color. The main modules were structured as follows:

Figure 5a illustrates the extraction of phenotypic features from longitudinal sections of tomato fruits, focusing on shape and stem scar features.

1. To extract morphological features from tomato sections, this study used image processing and threshold segmentation algorithms to generate a binary mask for each tomato fruit. A minimum bounding rectangle was fitted to each tomato fruit in the image, with its height defined as the longitudinal diameter and its width as the transverse diameter. The fruit shape index was calculated as the ratio between the longitudinal and transverse diameters of the fruit.
2. We utilized a SegFormer-MLLA model for tomato fruit phenotypic trait segmentation to achieve efficient and precise segmentation of the stem scar boundary. Based on the segmentation results, a minimum bounding rectangle was fitted to the stem scar, with its width and depth determined.
3. Using the obtained data, we integrated RGB-D information from the depth camera and employed the HDRM model to optimize depth values, thereby converting pixel distances into physical dimensions and obtaining the actual values of the tomato fruit’s transverse diameter, longitudinal diameter, stem scar depth, and stem scar width.

Figure 5b illustrates the extraction of phenotypic features from transverse sections of tomato fruits, emphasizing locule features, locule area, and mesocarp thickness.

1. Tomato fruit transverse section images were processed at 512 × 512 resolution. The SegFormer-MLLA model was applied to segment the locule structure for quantitative analysis of locule number and area. To ensure systematic and traceable analysis, a numbering system was designed for tomato fruits and their internal locules, assigning unique identifiers to establish correspondence.
2. Three rays were drawn from the centroid of each tomato fruit toward each locule. Experimental results showed that offsetting the two side rays by 12° from the central ray provided optimal performance. The minimum Euclidean distance between the intersection points of rays with the locule contour and the tomato outer contour was calculated, and their average was used as an approximate estimate of mesocarp thickness.
3. Using the depth information provided by the depth image and the HDRM model, pixel-based measurements of mesocarp thickness and locule area were converted into actual physical dimensions.
4. Further color recognition analysis was conducted. By averaging the RGB values of each tomato locule, the representative color features of the locule were obtained. Additionally, based on a tomato flesh mask (generated by subtracting the locule mask from the overall tomato mask), a morphological erosion algorithm was employed to extract the pericarp region near the tomato’s outer edge, and its color features were identified.

## 3. Results

### 3.1. Experimental Environment

The experiments were conducted on a system equipped with an NVIDIA GeForce RTX 4070 GPU and an Intel Core i5-13490F CPU, running the Windows 11 operating system. The integrated development environment was PyCharm 2024.2.2, and the compilation environment was Python 3.8. The MMSegmentation algorithm library was employed as the algorithmic framework. Input images were processed at a resolution of 512 × 512 pixels, using the AdamW optimizer with a learning rate of 1 × 10^−3^. The batch size was set to 4.

The imaging parameters of the Azure Kinect 3.0 depth camera were maintained at fixed values during data acquisition to ensure consistent illumination and color characteristics. Specifically, the brightness, contrast, saturation, and sharpness were set to 20, 50, 64, and 24, respectively. These values were obtained through tuning and calibration to achieve optimal visual clarity and depth stability under the controlled lighting conditions of the imaging environment.

Model training was conducted for 320,000 iterations for tomato stem scar segmentation and 240,000 iterations for tomato locule segmentation. The poly learning rate decay strategy was adopted, and the weight decay was 0.01. To address the class imbalance problem, particularly for small structures such as stem scars, a hybrid loss function combining Cross-Entropy Loss (weight = 0.7) and Dice Loss (weight = 0.3) was employed.

### 3.2. Evaluation Metrics

The segmentation performance of the SegFormer-MLLA model was evaluated using Intersection over Union (IoU), Dice coefficient, Accuracy, Precision, and Recall. IoU denotes the overlap between predicted and ground-truth regions. The Dice coefficient quantifies the boundary matching accuracy. Precision denotes the proportion of correctly predicted positive regions among all predicted positive regions. Recall denotes the proportion of correctly identified target regions relative to all actual target regions. The formulas for these four metrics are presented below:

(16) $$IoU=\frac{TP}{TP+FP+FN}$$

(17) $$Dice\;coefficient=\frac{2\times \left|X\cap Y\right|}{\left|X\right|+\left|Y\right|}=\frac{2TP}{2TP+FP+FN}$$

(18) $$Precision=\frac{TP}{TP+FP}$$

(19) $$Recall=\frac{TP}{TP+FN}$$

where *TP*, *FP*, and *FN* denote the number of true positives, false positives, and false negatives, respectively. *X* represents the segmented region predicted by the model, and *Y* indicates the ground-truth segmented region.

We evaluated the Hybrid Depth Regression Model using Root Mean Square Error (*RMSE*) to quantify its performance. Phenotypic traits of tomato fruit sections, including transverse and longitudinal diameters, mesocarp thickness, and stem scar depth and width, were manually measured using a vernier caliper. The fruit shape index was calculated as the ratio of longitudinal diameter to transverse diameter. To evaluate the performance of image-based dimension measurements, *RMSE*, *MAE*, and *R*^2^ were used as metrics:

(20) $$RMSE=\sqrt{\frac{1}{N}\sum_{i=1}^{N}{\left(y_{i-}{\overset{\wedge }{y}}_{i}\right)}^{2}}$$

(21) $$MAE=\frac{1}{N}\sum_{i=1}^{N}\left|y_{i-}{\overset{\wedge }{y}}_{i}\right|$$

(22) $$R^{2}=1-\frac{\sum_{i=1}^{N}{\left({\overset{\wedge }{y}}_{i}-y_{i}\right)}^{2}}{\sum_{i=1}^{N}{\left(y_{i}-\bar{y}\right)}^{2}}$$

where ${\overset{\wedge }{y}}_{i}$ denotes the predicted dimension, *y_i_* represents the ground-truth dimension, and *N* denotes the sample size.

### 3.3. Evaluation of Segmentation Results

To evaluate the performance of the SegFormer-MLLA model, comparative experiments were conducted under identical experimental conditions and test sets against several classical semantic segmentation algorithms, including UNet [44], DeepLabv3+ [45], PIDNet [46], Convnext [47], and Mask2Former [48]. The SegFormer model comprises multiple variants, such as B0 and B2, which differ in embedding dimensions and layer counts, representing lightweight and more complex architectures, respectively. In this study, SegFormer-MLLA-a and SegFormer-MLLA-b were proposed based, respectively, on the embedding dimensions and layer counts of the B0 and B2 models.

Table 1 shows the evaluation results for tomato stem scar segmentation. The lightweight SegFormer-MLLA-a model, with only 3.06 M parameters, achieved an IoU of 77.86%, comparable to the Mask2Former model’s IoU of 77.64% with 44.00 M parameters. Furthermore, the SegFormer-MLLA-b model, an enhanced version of the SegFormer-B2 architecture, achieved an IoU of 78.36% with a 23.2% parameter reduction (from 24.72 M to 18.98 M). It slightly outperformed the baseline model while maintaining stability in the Dice coefficient (87.87%) and Recall (87.59%). These results demonstrate the significant performance advantages of the SegFormer-MLLA model optimized with the MLLA Attention module.

Table 2 shows the evaluation results for tomato locules segmentation. As presented in Table 2, the SegFormer-MLLA model exhibited outstanding performance. The SegFormer-MLLA-a model, with only 3.06 M parameters, attained an IoU of 85.00%, representing a 0.16% improvement over the baseline SegFormer-B0, and increased the Dice coefficient to 91.88%. The SegFormer-MLLA-b model, with 18.98 M parameters, achieved an IoU of 85.24%, surpassing the Mask2Former model (IoU 85.15%) with 2.32 times the parameters. These results indicate that the MLLA module provides exceptional performance in fine-structure segmentation while maintaining high computational efficiency, highlighting the model’s effective balance between performance and resource demands.

### 3.4. Tomato Fruit Size Phenotypic Information Extraction

For the segmentation and dimension recognition tasks of tomato fruit section images, a strategy integrating preprocessing and depth information was employed. As original images contained multiple tomato targets with excessive background elements and small target sizes, training and prediction using unprocessed images led to reduced model segmentation accuracy. Therefore, an HSV color space segmentation combined with contour detection was initially applied to crop the original images into multiple 512 × 512 pixel single-tomato images, while the positional information of each tomato in the original image was recorded. Subsequently, semantic segmentation was performed on the cropped single-tomato images, and the segmentation results were recombined into the original image layout using the retained positional information. Ultimately, Precise measurements of the actual physical dimensions of tomato fruit sections were achieved by integrating RGB images with depth information. This approach effectively reduces background interference on segmentation accuracy, providing reliable support for depth vision-based tomato phenotyping.

**Figure 6:**
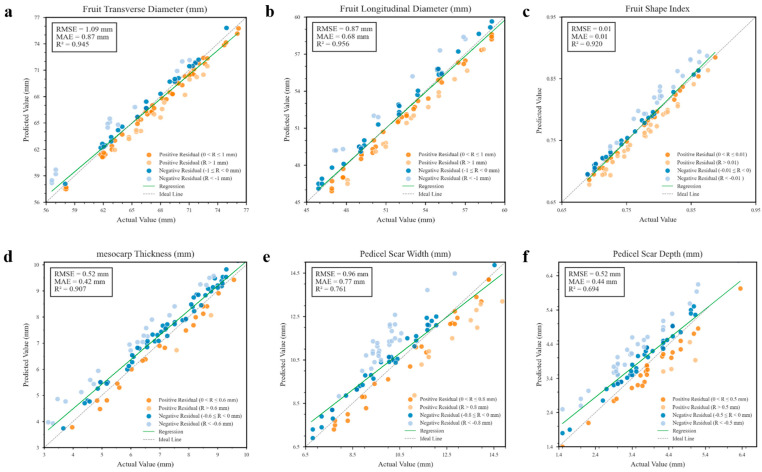
Tomato fruit phenotypic trait detection performance evaluation. (**a**–**f**) Assessment of phenotypic traits, including transverse diameter, longitudinal diameter, fruit shape index, mesocarp thickness, stem scar width, and depth.

The performance of the tomato fruit phenotyping intelligent detection framework was evaluated using 50 independent tomato samples imaged at two different heights, resulting in 100 independent sample sets in total. Benchmark values for fruit geometric parameters were obtained through manual measurements, and the fruit shape index was calculated. The phenotypic measurement results were compared with the corresponding predictions from the phenotypic detection model, depicted in Figure 6, which shows high consistency in the model’s predictions for transverse diameter, longitudinal diameter, and fruit shape index of tomato fruits. The RMSE values were 1.09 mm, 0.87 mm, and 0.01, respectively. The MAE values were 0.87 mm, 0.68 mm, and 0.01, respectively. The R^2^ values were 0.945, 0.956, and 0.920, respectively. For mesocarp thickness, the model achieved an RMSE of 0.52 mm, MAE of 0.42 mm, and R^2^ of 0.907.

**Figure 7:**
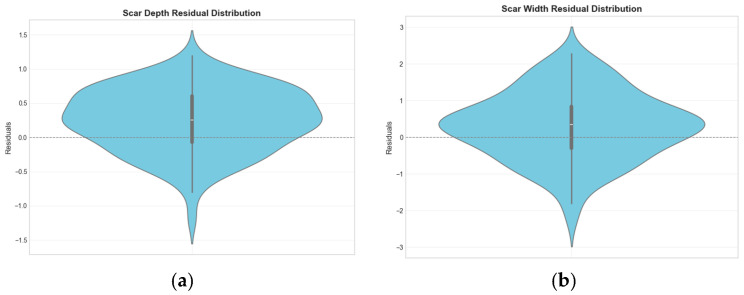
Residual distributions of fruit stem scar width (**a**) and depth (**b**).

Compared to the aforementioned parameters, the R^2^ value for the tomato fruit stem scar was slightly lower, attributable to its morphological diversity and the influence of segmentation outcomes. The violin plots in Figure 7, which illustrate the residual distributions for scar depth and width, provide evidence for this issue. In some varieties, the fruit stem scar area appears off-white or light yellow—a color similar to the placental tissue—resulting in unclear boundary recognition; hence, manual detection is recommended.

**Figure 8:**
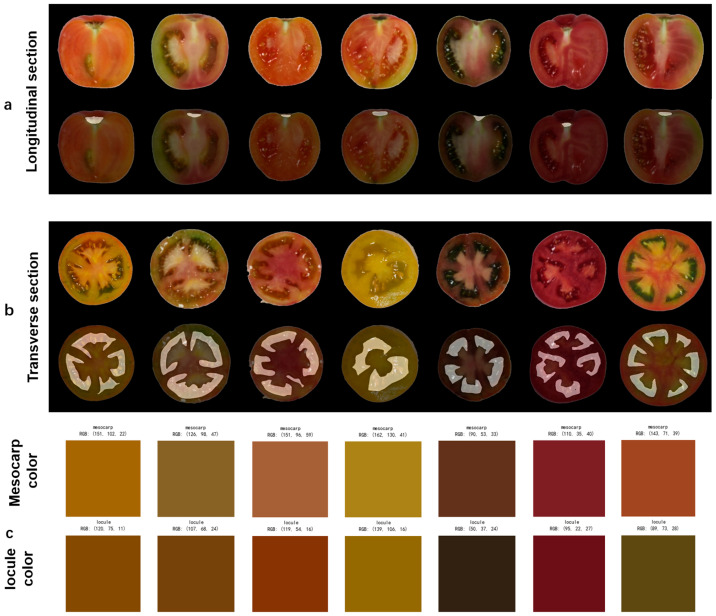
Detection results for tomato fruit. (**a**) Segmentation results for stem scar structure; (**b**) Segmentation results for locule structure; (**c**) Detection results for locule and mesocarp color.

Additionally, this study quantified color features by extracting RGB tri-channel values from the mesocarp and locules, illustrated in Figure 8, enabling a new dimension for assessing fruit maturity and internal structural characteristics. The color differences observed between the mesocarp and the locules in tomatoes are not merely visual but are intrinsically linked to the spatial distribution of key biochemical traits, particularly lycopene content [49]. The inclusion of color data enhances the diversity of phenotypic parameters and provides a data foundation for correlations between fruit quality and phenotypic traits. Compared to traditional manual measurements, this automated identification method enables rapid phenotyping of tomato fruits and effectively supports variety selection and quality grading in high-throughput agricultural phenotyping applications, significantly improving breeding screening efficiency.

### 3.5. Ablation Experiment

**Figure 9:**
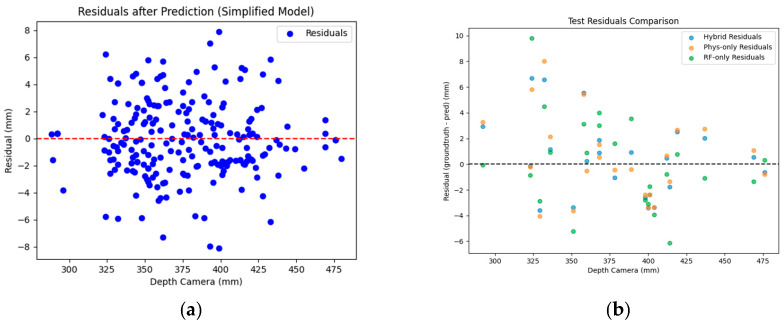
HDRM model detection performance evaluation. (**a**,**b**) HDRM model assessment through residual scatter plot and comparison of predicted versus ground-truth depth values.

Building on the SegFormer-MLLA model for tomato fruit structure segmentation, this study extracted 10 key phenotypic parameters from RGB-D images of fruit sections. The precision issue in size measurement arises from depth value bias in depth cameras. A Hybrid Depth Regression Model (HDRM) was introduced to correct the depth values, effectively improving measurement accuracy. A total of 264 paired samples were used, divided into training and testing sets with a 9:1 ratio, and evaluated through ten-fold cross-validation to ensure model robustness. As shown in Figure 9, the optimized depth measurements significantly reduced the RMSE from 18.754 mm (original) to 3.011 mm using the proposed HDRM, compared to 3.154 mm for the parameterized modeling approach and 3.500 mm for the Random Forest model.

To evaluate the performance of different modeling strategies in predicting fruit morphological parameters, the predictive accuracy of the Random Forest model, the parametric model, and the HDRM (combining both approaches) was compared. The results in Table 3 show that the Random Forest model can capture nonlinear relationships but tends to exhibit bias when the dataset is small or lacks sufficient constraints. The parametric model provides good interpretability but has limited ability to fit systematic errors. In contrast, the proposed HDRM achieved the best performance across all metrics, with RMSE values for transverse diameter, longitudinal diameter reduced to 1.064 mm and 0.956 mm.

Furthermore, the contribution of each component within the overall framework was evaluated through an ablation study, as presented in Table 4. When employing the Segormer-MALL, the model achieved strong detection performance, with the introduction of the MLLA module enhancing its ability to represent structural details of tomato locules and stem scars. In the depth correction component, using the Random Forest model alone exhibited a certain degree of instability, whereas the parametric modeling approach effectively reduced systematic errors. By integrating both methods, the HDRM mitigated fitting bias and improved prediction stability. The complete framework achieved the lowest RMSE and MAE across most evaluation metrics.

### 3.6. Device Detection and Software Development

**Figure 10:**
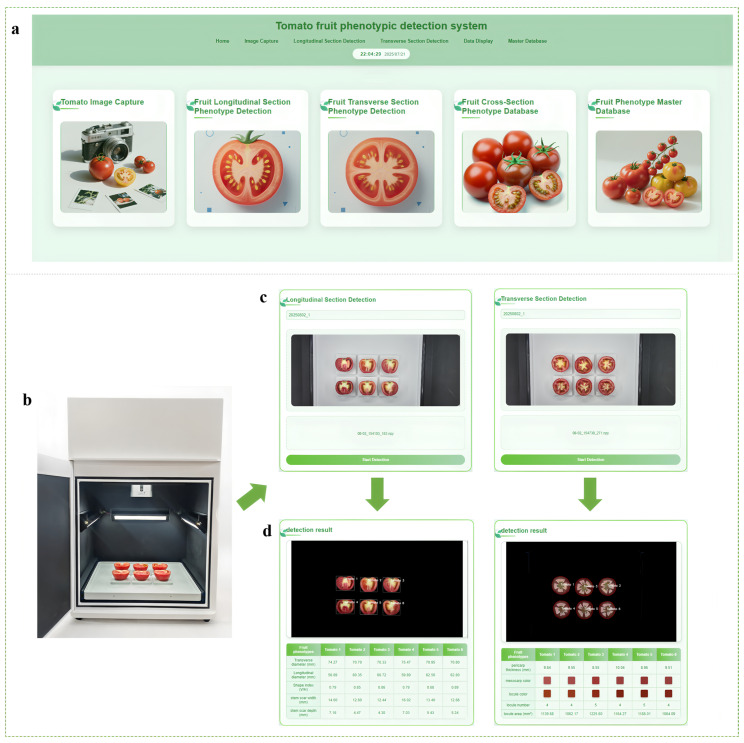
Schematic diagram of the automated detection equipment and software for tomato fruit phenotyping. (**a**) Main functional modules of the detection system; (**b**) Structural diagram of the equipment; (**c**) Detection process for longitudinal and transverse sections of tomato fruit phenotypes; (**d**) Results of tomato fruit phenotype detection.

To automate the extraction and quantitative analysis of tomato fruit phenotypic traits, we integrated multiple algorithms and techniques, including image processing, semantic segmentation, and regression models. Using these methods, we successfully designed and implemented an efficient automated detection equipment for tomato fruit phenotyping, along with its supporting software, as shown in Figure 10. The equipment primarily consists of the following components: an Azure Kinect depth camera, a sample tray for the stable placement of fruits (capable of holding six tomatoes), LED light strips to provide a uniform and controllable lighting environment, and a sealed light-shielded imaging box to isolate ambient light. The accompanying software provides a user-friendly interface and integrates core functions, including real-time capture, processing, and storage of RGB-D images, automatic extraction and quantitative analysis of tomato fruit phenotypic parameters, and the creation of a phenotypic database for structured storage and management of tomato fruit data. This device serves as a laboratory tool designed for precise internal phenotyping of tomato fruits obtained from breeding and cultivation experiments.

## 4. Discussion

Amid the rapid advancement of intelligent breeding technologies, efficiently and accurately extracting phenotypic information from tomato fruits remains a key challenge. Because traditional manual phenotyping measurement methods are time-consuming and labor-intensive, they are not suitable for the high-throughput, large-sample demands of modern breeding. In the field of automated tomato fruit analysis, there has been considerable research on external quality attributes [50,51,52], while studies on intelligent recognition of internal structures remain relatively limited. This study focuses on methodological research for the intelligent acquisition of internal phenotypic traits in tomato fruits. We obtained internal phenotypic traits such as locule number, mesocarp thickness, and stem scar morphology by performing both transverse and longitudinal sectioning of tomato fruits. These destructive measurements are necessary to access internal structures that cannot be observed non-invasively. Although the method is inherently destructive, it enables the acquisition of precise and detailed internal phenotypic data at a low cost. Furthermore, the enclosed imaging box ensures stable and uniform illumination, effectively minimizing ambient light interference and guaranteeing consistent imaging quality across samples. Accordingly, the accuracy reported in this study is specific to the conditions used: the analysis of sectioned fruits within an enclosed imaging system. The results are therefore not directly applicable to intact fruits in open environments.

In tomato fruit phenotypic size detection, most methods typically rely on a ruler or black-and-white scale card as a reference for measuring fruit-related phenotypic traits [30,31,53], which require the reference object and the fruit to be positioned on the same focal plane and aligned with the fruit’s primary measurement axis; otherwise, substantial measurement errors may arise. In this study, a method that combined RGB images with depth information was employed for the accurate detection of tomato fruit phenotypic size, eliminating the need for a black-and-white scale card, and enabling high-throughput measurement across large sample sets. This approach can be extended to other crops with similar fruit characteristics, requiring minor fine-tuning and recalibration to accommodate differences in tissue structure and phenotypic traits.

### 4.1. The Feasibility and Practical Significance of SegFormer-MLLA in Tomato Fruit Trait Analysis

With the increasing demand for automated analysis of tomato fruit traits, efficient utilization of computational resources in resource-constrained environments is critical for real-time or near-real-time applications [54]. To address the challenge of automated and precise phenotyping of complex tomato fruit traits, we propose a novel lightweight segmentation model, SegFormer-MLLA, for efficient locule and stem scar segmentation, meeting the demands of resource-constrained environments. The segmentation results generated by this model further enable the automatic extraction of multiple key phenotypic parameters, such as tomato mesocarp thickness and stem scar characteristics. Previous research [29] utilized the Mask R-CNN model to extract tomato fruit phenotypes, but it exhibited limitations in model efficiency. The model proposed in this study has only 3.06 M parameters, offering higher efficiency and greater suitability for scalable deployment.

### 4.2. Effect of HDRM Model on Size Detection

During the experiment, we observed deviations in the restoration of fruit phenotypic dimensions using depth information. This deviation is likely due to light absorption by moisture in the tomato fruit’s cross-sectional tissue and light reflection caused by its smooth surface. To overcome this limitation, a Hybrid Depth Regression Model (HDRM) is used to optimize the captured depth values, which, together with the camera’s intrinsic matrix, enables conversion of pixel measurements in RGB images into the fruit’s actual physical dimensions. Evaluated using R^2^, RMSE, and MAE metrics, our method achieved high accuracy in measuring tomato phenotypic traits, including transverse diameter, longitudinal diameter, mesocarp thickness, and fruit shape index.

### 4.3. Phenotypic Research of Tomato Stem Scars

Currently, significant progress has been made in rapid phenotyping of vegetables such as tomatoes, but research has primarily focused on external fruit traits, such as size and shape, while studies on internal structures and localized features, such as stem scars, remain scarce. The tomato stem scar is a wound formed when the stem separates from the fruit during harvesting. Its rapid healing forms a hydrophobic barrier, which helps reduce post-harvest water loss and microbial infection, thereby extending the storage period and thus its importance in tomato post-harvest handling [55]. Based on our method, the extraction and segmentation of stem scars were successfully achieved, with dimensional detection errors controlled at the sub-millimeter level, providing significant advantages for automated phenotypic analysis in breeding programs.

### 4.4. Benefits of the Automated Phenotyping System

To apply our established models and methods more efficiently to the systematic analysis of tomato fruit phenotyping, we built on the intelligent detection framework and developed automated phenotyping equipment and software. This system effectively eliminates subjective errors in manual measurements, enhances detection convenience, significantly improves the consistency and reliability of phenotypic data, and lowers the operational threshold. Furthermore, we established a comprehensive tomato phenotyping database, enabling long-term storage, traceability, and reuse of detection data and images, which facilitates comparative analysis, model training, and data sharing, providing critical support for genotype-phenotype association studies.

### 4.5. Limitations and Future Work

Although this study has made progress in the automated detection of tomato fruit phenotypic traits, certain limitations remain.

During fruit size detection, slight angular deviations between the tomato fruit section plane and the camera may occur, even if visually undetectable. These deviations can lead to non-uniform depth value distribution across the plane, introducing measurement errors. As shown in Table 5, our method demonstrates high accuracy in measuring tomato fruit phenotypic traits by comparing the true and detected values of multiple tomato samples at six equipment positions. Slight variations in the detection outcomes were primarily due to changes in orientation or visual perspective. For instance, a 5° tilt in the tomato fruit section resulted in a deviation of approximately 2–3 mm in the depth value.

To address these challenges, our research will focus on key directions to advance tomato phenomics. First, we will refine our model and expand training datasets to include a broader range of tomato cultivars, thereby enhancing the accuracy and robustness of detecting tomato fruit phenotypic traits. Second, we plan to explore the application of point cloud, spectral, and three-dimensional technologies in tomato phenotyping, aiming to develop innovative approaches to study more phenotypes, thereby enhancing the precision and scope of trait analysis in agricultural research. Third, we will implement equipment enhancements, such as replacing the fixed tray for holding tomato fruits with a conveyorized system to mitigate angular deviations between the fruit section plane and the camera.

## 5. Conclusions

In this study, an intelligent detection framework for tomato fruit phenomics analysis was developed, which combines image processing techniques with deep learning algorithms to automate the extraction and quantitative analysis of 12 phenotypic traits. First, a dataset of tomato fruit section images was developed using a depth camera. Second, a lightweight SegFormer-MLLA model for tomato fruit phenotype segmentation was proposed. Accurate segmentation of tomato fruit stem scars and locular structures was achieved, with significantly reduced computational cost by the proposed model. Finally, an HDRM model was designed to optimize the estimation of optimal depth. Building on this, we developed an automated phenotyping system for tomato fruits, which provides a controlled and stable environment to enable precise trait detection. The results showed that the RMSEs were approximately 1 mm for the longitudinal diameter, transverse diameter, and stem scar width of the tomato fruit sections, and below 0.6 mm for the mesocarp thickness and stem scar depth.

## Figures and Tables

**Table 1:** Evaluation results for tomato stem scar segmentation.

Models	Stem Scar				
	IoU (%)	Dice (%)	Precision (%)	Recall (%)	Parameters (M)
Unet	74.52	85.32	84.97	85.26	29.06
Deeplabv3+	74.78	85.41	85.73	85.41	43.59
Pidnet	73.37	84.64	85.14	84.15	7.72
Convnext	76.95	86.97	87.96	86.01	59.28
Mask2former	77.64	89.12	85.76	89.12	44.00
SegFormer-b0	77.82	87.46	87.34	87.58	3.72
SegFormer-MLLA-a	77.86	87.55	87.59	87.51	3.06
SegFormer-b2	78.29	87.82	88.44	87.21	24.72
SegFormer-MLLA-b	78.36	87.87	88.15	87.59	18.98

**Table 2:** Evaluation results for tomato locules segmentation.

Models	Locule				
	IoU (%)	Dice (%)	Precision (%)	Recall (%)	Parameters (M)
Unet	84.26	91.47	92.02	90.93	29.06
Deeplabv3+	84.43	91.56	92.03	91.10	43.59
Pidnet	84.33	91.50	92.27	90.74	7.72
Convnext	84.62	91.67	91.46	91.88	59.28
Mask2former	85.15	91.98	92.48	91.49	44.00
SegFormer-b0	84.84	91.80	92.33	91.27	3.72
SegFormer-MLLA-a	85.00	91.88	91.88	91.91	3.06
SegFormer-b2	85.04	91.92	92.57	91.27	24.72
SegFormer-MLLA-b	85.24	92.03	92.47	91.59	18.98

**Table 3:** Performance comparison of different modeling strategies for predicting fruit morphological parameters.

HDRM		Transverse Diameter (mm)		Longitudinal Diameter (mm)	
Random Forest	Parametric Model	*RMSE*	*MAE*	*RMSE*	*MAE*
		2.312	2.176	2.824	2.600
√		1.153	0.946	0.991	0.818
	√	1.072	0.891	0.970	0.808
√	√	1.064	0.880	0.965	0.803

The symbol √ indicates that the corresponding module was used

**Table 4:** Ablation Study Results of the Intelligent Tomato Fruit Detection Framework.

SegFormer	MLLA	HDRM		Mesocarp Thickness		Stem Scar Width		Stem Scar Depth	
		Random Forest	Parametric Model	*RMSE*	*MAE*	*RMSE*	*MAE*	*RMSE*	*MAE*
√				0.682	0.586	1.163	0.957	0.553	0.457
√	√			0.686	0.527	1.147	0.944	0.473	0.371
√		√		0.484	0.378	0.990	0.740	0.447	0.367
√	√	√		0.371	0.322	0.983	0.777	0.441	0.360
√			√	0.463	0.355	0.998	0.780	0.449	0.367
√	√		√	0.348	0.304	0.940	0.735	0.399	0.319
√		√	√	0.458	0.353	0.986	0.772	0.447	0.367
√	√	√	√	0.349	0.303	0.937	0.735	0.397	0.315

The symbol √ indicates that the corresponding module was used

**Table 5:** Comparison of Measured and Detected Phenotypes of Tomato Fruits Across Multiple Positions.

Phenotypic Trait	Measure Size	Position					
		1	2	3	4	5	6
transverse diameter (mm)	74.91	74.61	73.36	75.17	74.28	73.20	74.48
longitudinal diameter (mm)	59.86	59.91	59.15	59.68	59.85	59.41	59.35
shape index	0.80	0.80	0.81	0.79	0.81	0.81	0.80
stem scar width (mm)	13.23	13.61	13.16	13.34	13.61	13.27	12.91
stem scar depth (mm)	4.67	4.64	5.13	4.99	5.28	5.32	5.31
mesocarp thickness (mm)	8.36	8.63	8.28	8.42	8.45	8.30	8.47
locule number	6	6	6	6	6	6	6

## Data Availability

Some datasets, model weights, and code used in the present study are available at https://github.com/Snail-code-wq/Plants_Tomato_2025 (accessed on 5 November 2025). All self-developed datasets can be obtained by contacting the corresponding author.
